# Chronic administration of the probiotic kefir improves the endothelial function in spontaneously hypertensive rats

**DOI:** 10.1186/s12967-015-0759-7

**Published:** 2015-12-30

**Authors:** Andreia G. F. Friques, Clarisse M. Arpini, Ieda C. Kalil, Agata L. Gava, Marcos A. Leal, Marcella L. Porto, Breno V. Nogueira, Ananda T. Dias, Tadeu U. Andrade, Thiago Melo C. Pereira, Silvana S. Meyrelles, Bianca P. Campagnaro, Elisardo C. Vasquez

**Affiliations:** Pharmaceutical Sciences Graduate Program, Vila Velha University, Vila Velha, ES Brazil; Division of Nephrology, McMaster University, Hamilton, ON Canada; Laboratory of Translational Physiology, Federal University of Espirito Santo, Vitoria, ES Brazil; Department of Morphology, Federal University of Espirito Santo, Vitoria, ES Brazil; Institute of Education, Science and Technology (IFES), Vila Velha, ES Brazil

**Keywords:** Kefir, Probiotics, Spontaneously hypertensive rat, Oxidative stress, Endothelial dysfunction

## Abstract

**Background:**

The beverage obtained by fermentation of milk with kefir grains, a complex matrix containing acid bacteria and yeasts, has been shown to have beneficial effects in various diseases. However, its effects on hypertension and endothelial dysfunction are not yet clear. In this study, we evaluated the effects of kefir on endothelial cells and vascular responsiveness in spontaneously hypertensive rats (SHR).

**Methods:**

SHR were treated with kefir (0.3 mL/100 g body weight) for 7, 15, 30 and 60 days and compared with non-treated SHR and with normotensive Wistar-Kyoto rats. Vascular endothelial function was evaluated in aortic rings through the relaxation response to acetylcholine (ACh). The balance between reactive oxygen species (ROS) and nitric oxide (NO) synthase was evaluated through specific blockers in the ACh-induced responses and through flow cytometry in vascular tissue.

**Results:**

Significant effects of kefir were observed only after treatment for 60 days. The high blood pressure and tachycardia exhibited by the SHR were attenuated by approximately 15 % in the SHR-kefir group. The impaired ACh-induced relaxation of the aortic rings observed in the SHR (37 ± 4 %, compared to the Wistar rats: 74 ± 5 %), was significantly attenuated in the SHR group chronically treated with kefir (52 ± 4 %). The difference in the area under the curve between before and after the NADPH oxidase blockade or NO synthase blockade of aortic rings from SHR were of approximately +90 and −60 %, respectively, when compared with Wistar rats. In the aortic rings from the SHR-kefir group, these values were reduced to +50 and −40 %, respectively. Flow cytometric analysis of aortic endothelial cells revealed increased ROS production and decreased NO bioavailability in the SHR, which were significantly attenuated by the treatment with kefir. Scanning electronic microscopy showed vascular endothelial surface injury in SHR, which was partially protected following administration of kefir for 60 days. In addition, the recruitment of endothelial progenitor cells was decreased in the non-treated SHR and partially restored by kefir treatment.

**Conclusions:**

Kefir treatment for 60 days was able to improve the endothelial function in SHR by partially restoring the ROS/NO imbalance and the endothelial architecture due to endothelial progenitor cells recruitment.

## Background

Endothelial cells play a central role in the maintenance of the vascular homeostasis and endothelial dysfunction has been considered to be an important characteristic that accompanies states of metabolic diseases and arterial hypertension [1–4]. Most of the studies related to endothelial dysfunction have been performed with spontaneously hypertensive rats (SHR), which exhibit impaired endothelium-dependent relaxation attributable to excessive generation of NADPH oxidase-driven reactive oxygen species (ROS) and decreased nitric oxide (NO) bioavailability [5, 6]. Thus, SHR have been used as an important tool for the understanding of hypertension and endothelial dysfunction as well as for the identification of alternative or non-pharmacological agents for prevention/treatment of these diseases.

Previous studies have demonstrated the beneficial effects of functional foods, which exhibit several health promoting properties in experimental and clinical cardiovascular studies [7], including the decrease in blood pressure in mildly hypertensive subjects [8] and SHR chronically treated with these products [9]. Among the varieties of scientifically validated functional foods available, an important focus of the investigations have been fermented milks containing lactic acid bacteria, the so-called probiotics, including kefir. The alleged health-promoting characteristics of kefir have been reviewed [10]. This food product originated in the Northern Caucasus has been distributed worldwide. While in some countries kefir is already commercially available [11], in our country, this probiotic has traditionally been distributed person-to-person and it has been domestically produced by using kefir grains as a starter [12]. Milk fermentation with kefir grains forms a matrix made up of polysaccharides and proteins primarily produced by the lactic acid bacteria and yeast species, which are a complex microbial symbiotic relationship [13] that results in the production of biogenic elements [12, 13].

The effects of isolated microorganisms from probiotics on blood pressure in hypertensive subjects [14] and in experimental models of hypertension [15, 16] have been investigated. Some studies have investigated their effects on the endothelial dysfunction that accompanies arterial hypertension and other cardiovascular diseases [16]. However, the effects of the beverage obtained by fermentation of milk with kefir grains, on the endothelial dysfunction observe in the context of hypertension have not yet been investigated.

Therefore, our study was designed to test the hypothesis that chronic treatment with kefir may have beneficial effects on high blood pressure and endothelial dysfunction in SHR, a classical model for essential hypertension. The importance of the present study is highlighted by the fact that it evaluated the effects of kefir grains, which contain a relatively stable and specific microbiota enclosed in a matrix of polysaccharides and proteins [17]. The present data revealed new insights on the time-dependent actions of kefir on functional and structural endothelial abnormalities. The findings of the present study included the beneficial effects of kefir on the imbalance between ROS production and NO bioavailability and the recruitment of endothelial progenitor cells (EPC) to repair the damage to the endothelial surface layer in the SHR.

## Methods

### Animals

The present study was performed in male 4-month-old SHR and in age-matched Wistar-Kyoto rats, both of which were maintained in the animal care facility of the Federal University of Espirito Santo, Brazil. The rats were acclimatized and housed in individual plastic cages with a controlled temperature (22–23 °C) and light–dark cycle (12:12 h) and were fed with a standard rat chow and provided with water ad libitum. The study protocols were approved by the Institutional Committee on Animal Care (CEUA, Protocol #040/2014). All experimental procedures were performed in accordance with the guidelines for the care and use of laboratory animals as recommended by the National Institutes of Health (NIH).

### Kefir: identification, preparation and administration

The kefir used in the present study was obtained from the fermentation of the grains in whole milk, as commonly consumed by Brazilian people. The bacteria and yeasts were identified by the surface spread technique using four different Agar media (Acetobacter, Nurient, MRS and Sabouraud) and specific conditions of temperature (25, 30 and 37 °C), atmosphere (aerobiosis and anaerobiosis) and time (24, 48, 96 and 120 h). Subsequently, the bacterial isolates were Gram-stained and examined for colony and cell appearance and for catalase, oxidase, coagulase and bile-esculin activity. After isolation of the strains, the species were confirmed by using the API galleries (BioMérieux, France).

The kefir beverage was prepared by adding kefir grains to pasteurized whole milk in a ratio of 4 % (w/v) and kept at room temperature. After 24 h, this mixture was filtered through a plastic screen and the resultant product was refrigerated (averaging 10 °C) to permit yeast growth for 24 h. At the end of this process, the kefir was aliquoted into sterile plastic tubes and stored at −20 °C until use.

One group of the SHR was treated with kefir (0.3 mL/100 g body weight, by gavage) and subdivided into 4 different groups, according with the duration of the treatment, i.e., for 7, 15, 30 and 60 days. The other group of SHR was administered whole milk (0.3 mL/100 g body weight, pH adjusted to 5.0) for 7–60 days for use as the hypertensive controls. The Wistar rats were administered whole milk for 7–60 days and were used as normotensive control groups.

### Instrumentation for hemodynamic measurements

After 7–60 days of kefir administration, the animals were intraperitoneally anesthetized with ketamine plus xylazine (91 + 9.1 mg/kg) and a polyethylene catheter (PE 50) filled with heparinized saline (40 U/mL) was positioned into the inferior aorta through the left femoral artery and exteriorized to the back of the neck. After 48 h, the end of the arterial catheter was attached to an external line that was attached to a disposable blood pressure transducer connected to a pressure processor amplifier and a data-acquisition system (Biopac Systems, Santa Barbara, CA, USA) for measurement of mean arterial pressure and heart rate in unrestrained animals after a period of 30 min of stabilization. The average of three successive measurements was taken as the mean arterial pressure value.

### Analysis of vascular function

Following treatment, the rats were anesthetized as above and a midline abdominal incision was performed to expose and isolate the thoracic aorta, which was carefully cleaned of the adherent connective tissue under a light microscope, and cut into 3–4 mm rings. Each aortic ring was mounted on stainless steel triangles, suspended vertically in tissue chambers containing 5 mL of modified Krebs buffer (composition in mmol/L: NaCl 119.0, NaHCO_3_ 25.0, glucose 11.2, CaCl_2_ 1.6, KCl 4.7, KH_2_PO_4_ 1.2, MgSO_4_ 1.2). The aortic rings were then allowed to equilibrate at an optimal tension of 1 g for 60 min at 37 °C and were continuously oxygenated with a mixture of 95 % O_2_ and 5 % CO_2_. The Krebs solution was replaced every 30 min, and the tension on each aortic ring was readjusted to 1 g when necessary. The changes in tension during the protocol were recorded isometrically using a force–displacement transducer connected to a computerized data acquisition system (Biopac Systems Inc.).

The time-course of the endothelium-dependent relaxation was tested after a washout period of 30 min using response curves to cumulative concentrations of ACh (10^−11^ to 10^−4.5^ mol/L) in aortic rings pre-contracted with phenylephrine (PE, 10^−6^ mol/L). We challenged the aortic rings with ACh at those concentrations because, in male SHR, concentration of ACh equal or higher than 10^−4^ mol/L results in contractions instead of relaxations. For each curve, the maximum effect (Rmax; the upper plateau of the sigmoidal curve) and the log of the concentration of the agonist that produced half of Rmax (log EC_50_) were calculated using nonlinear regression analysis and the sensitivities of the agonists were expressed as pEC_50_ (−log EC_50_). The vasorelaxation response to ACh was expressed as the percentage of vasodilation relative to the maximal PE-induced pre-contraction level.

The role of NO in the relaxation response to ACh was evaluated through pre-incubation of the aortic rings with the non-specific NO synthase (NOS) inhibitor N(G)-nitro-l-arginine methyl ester (L-NAME, 100 μmol/L) for 20 min. The differences in the area under the curves (ΔAUC) for the responses of the aortic rings before and after the presence of the inhibitor were calculated and these results were expressed in arbitrary units (a.u.). In a separate set of experiments, the role for ROS in the relaxation response to ACh as a function of time was evaluated by incubation of the aortic rings with the NADPH oxidase inhibitor apocynin (30 μmol/L), which was added to the vessel bath 20 min prior to assessing the dose–response curves to ACh. The ΔAUC, Rmax and the pEC_50_ were calculated. In a third set of experiments, the endothelium-independent relaxation was tested using sodium nitroprusside (SNP, 10^−10^ to 10^−5^ mol/L), a donor of NO. To address the contribution of the basal NO/cGMP pathway to the relaxation response of the aortic rings to ACh in PE-induced pre-constricted aortic rings, we evaluated the responses to ACh after inhibition of endothelial NO synthase with L-NAME (100 μmol/L).

### Scanning electron microscopy

Scanning electron microscopy was performed following modified methods as previously described [18]. Briefly, in separate subgroups of animals at time-point of 60 days, the entire thoracic aorta was carefully removed and dissected free of connective tissue, fixed in 0.1 mol/L Karnovsky-cacodylate buffer (solution A; pH 7.2) for 24 h, and post-fixed in a solution of 1.0 % osmium tetroxide, 1.25 % potassium ferrocyanide and 0.2 mol/L cacodylate buffer (solution B) for 1 h,. The samples were then washed in cacodylate buffer (0.1 mol/L) and ultrapure water and cut open, in longitudinal sections under a stereomicroscope, dehydrated in ascending grades of ethanol, and critical-point dried with liquid CO_2_. The specimens were mounted on stubs sputter coated with 10 nm of pure gold and examined using a scanning electron microscope (Jeol, JEM6610 LV, Jeol Inc., USA). For each specimen, four photomicrographs were randomly taken at × 1000 and × 3000 magnification.

Brazilian kefir grains were also analyzed by scanning electron microscopy, as previously described by Magalhães et al. [12]. Briefly, after the preparation of kefir, the grains were fixed in Solution A for 48 h, post-fixed in Solution B for 1 h, dehydrated in ascending grades of ethanol, critical-point dried and coated with gold. The preparations were observed using the scanning electron microscopy.

### Flow cytometry analysis of endothelial cells

Flow cytometry was performed using a FACS Canto II (Becton–Dickinson, BD, CA, USA) instrument to quantify the endothelial cells and analyze the intracytoplasmic ROS content. For endothelial cell counting, the animals were anesthetized as above and the aortic arch was isolated, minced and digested using type II collagenase (1000 U/mL, at 37 °C for 60 min at constant shaking). The tissue fragments were removed by filtration using a sterile 70-μm nylon mesh. The free cells were immediately washed twice in PBS, to remove the excess collagenase, and the cell suspension was stored at −80 °C. The cryovials from each animal were thawed in a 37 °C heated orbital shaker and slowly diluted into 5 mL of DMEM containing 20 % FBS. The numbers of endothelial cells in the aortas were determined using an APC-conjugated monoclonal antibody against platelet endothelial cell adhesion molecule (CD31-APC). Briefly, 1 × 10^5^ cells were re-suspended in PBS and incubated with 5 μL of CD31-APC or the respective isotype-matched APC-conjugated control antibody in the dark (20 min, RT). From each sample, 100,000 events were acquired and processed using the FACS Diva software (Becton–Dickinson, BD, CA, USA).

The intracytoplasmic ROS content was determined in isolated endothelial cells, as previously described (3). Intracellular •O_2_^−^, H_2_O_2_, ONOO^−^/•OH^−^ and NO were monitored separately by measuring changes in median fluorescence intensity (MFI) emitted by dihydroethidine (DHE), dichlorofluorescein (DCF), hydroxyphenyl fluorescein (HPF), and diaminofluorescein (DAF), respectively. Briefly, 10^6^ cells were incubated with 160 mmol/L of DHE, 20 mmol/L of DCF, 10 μmol/L of HPF, or 2 μmol/L of DAF at 37 °C for 30 min (DHE, DCF and HPF) or 180 min (DAF) in the dark. The samples were then washed, resuspended in PBS and kept on ice until the acquisition of 100,000 events by flow cytometry, which were subsequently analyzed using FACS Diva software.

### Quantification of circulating endothelial progenitor cells (EPC)

Flow cytometry was performed to quantify and characterize the phenotypes of the circulating EPC. After collection of whole blood and removal of erythrocytes, the samples were purified by negative selection with monoclonal antibodies against CD3e (CD3 ε chain), CD11b (Integrin αM chain), CD45R/B220, Ly-6G and Ly-6C (Gr-1), and TER-119/Erythroid Cells (Ly-76) (BD Biosciences, San Diego, CA, USA) for 15 min on ice, to deplete the lineage committed cells. Subsequently, the cells were magnetically labeled, loaded into a BD IMagnet column (BD), and the depleted cell fraction (Lin^−^) was carefully collected and analyzed by flow cytometry for quantification of the circulating EPC. The cell aliquots (1 × 10^6^ cells/mL of PBS) were stained for EPC immunophenotyping using anti-rat CD117-PECy7 and CD31-APC (BD) for 20 min in the dark. From each sample, 100,000 events were acquired (FACS Canto II, BD Bioscience) and processed using the FACS Diva software (Becton–Dickinson, BD, CA, USA). The circulating EPC were defined as Lin negative, CD117/CD31- double-positive cells (Lin^−^/CD117^+^/CD31^+^).

### Statistical analysis

The values are expressed as the mean ± S.E.M. Endothelium-dependent and independent smooth muscle vascular relaxation to cumulative concentrations of ACh or SNP, respectively, were analyzed by fitting a logistic function. The maximum effect (Rmax) and the logarithm of the molar concentration of the agonist that produced half of the Rmax (log EC_50_) were calculated. The sensitivity to ACh or SNP was estimated using the pEC_50_ (−log EC_50_). The statistical comparisons between the different groups were performed by either one-way or two-way analysis of variance (ANOVA) for repeated measures or completely randomized, followed by Bonferroni’s post hoc test. A value of p < 0.05 was considered statistically significant. The Statistical analyses were performed using GraphPad Prism software version 6.07.

## Results

### Identification of microbial isolates

The microbiological analysis of random samples of the grains used in this study showed that the dominant microflora of kefir included various beneficial bacteria (*Acetobacter aceti, Acetobacter* sp.*, Lactobacillus delbrueckii delbrueckii, Lactobacillus fermentum, Lactobacillus fructivorans, Enterococcus faecium, Leuconostoc* spp.), as well as *Lactobacillus kefiranofaciens,* and yeasts (*Candida famata, Candida krusei*). In another investigation in our laboratory, the microbiological analysis of kefir grains revealed that the global counting of microorganisms was 7.5 × 10^7^ CFU/mL.

### Microscopy of kefir grains

**Fig. 1:**
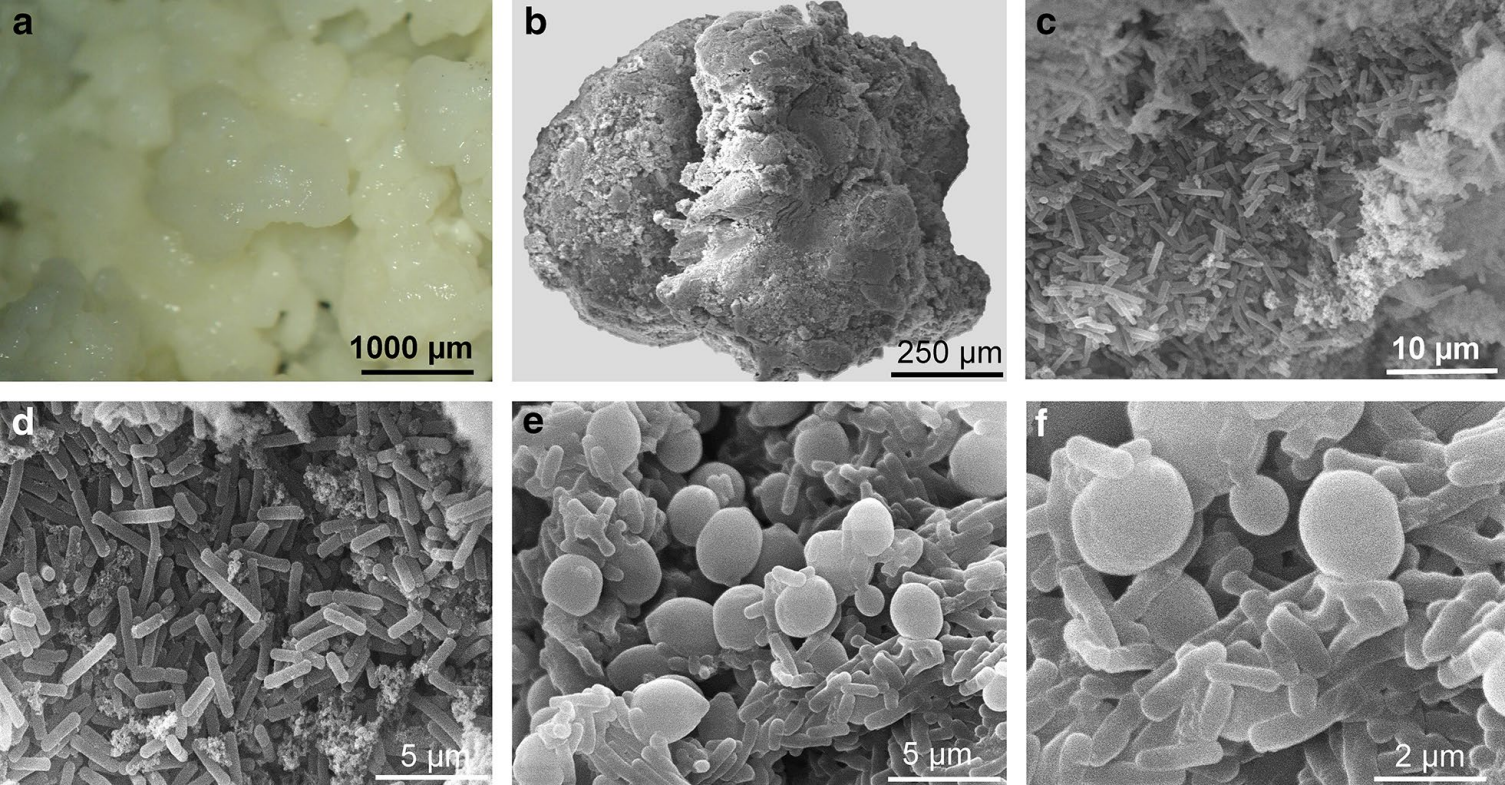
Photomicrographs of kefir grains obtained at the unmagnified level (**a**) and scanning electronic micrographs of the exterior (**b**–**d**) and interior (**e**, **f**) surface of a kefir grain. The external surface (**c**, **d**) shows the prevalence of the bacilli in tight association with a polysaccharide matrix (kefiran). The inside surface shows* rod-shaped* bacilli growing in association with yeasts (**e**, **f**)

Figure 1a shows photomicrographs at low magnification and without magnification of a sample of the gelatinous white kefir grains that were used to ferment the milk in our study. Figure 1b–e are representative photomicrographs of kefir grains obtained using scanning electronic microscopy showing high-magnifications images of the outside (b–d) and inside (e, f) surface of kefir grains. The images generated by scanning electronic microscopy show a tightly associated agglomerate of various microorganisms around the kefir grains. The exterior surface (c, d) shows that the prevalent forms of the microorganisms in the kefir biofilm were short and long curved bacilli in tight association with a polysaccharide matrix (Fig. 1d). The interior surface suggests that they grow in association with the ovoid-shaped yeast cells (Fig. 1e, f).

### Kefir improves blood pressure and heart rate

**Fig. 2:**
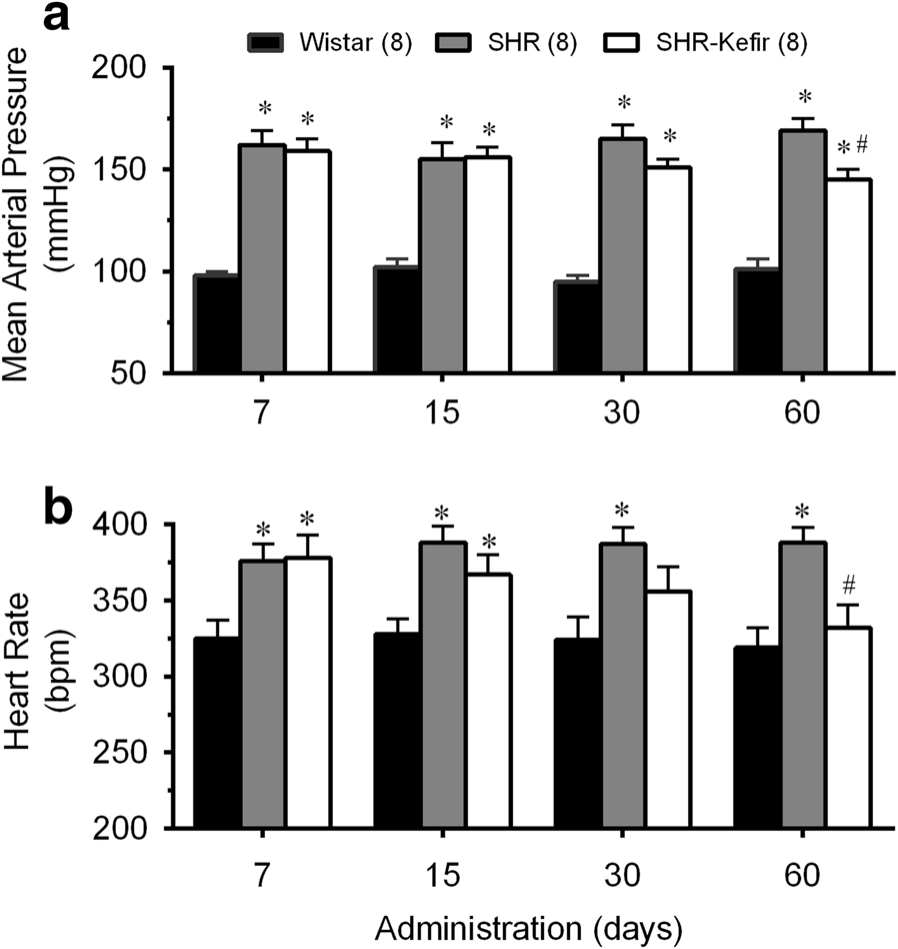
Time-course of the effects of kefir on hemodynamic parameters in SHR. The bar graphs show average values (mean ± SEM) of the mean arterial pressure (**a**) and heart rate (**b**) of SHR treated with kefir compared to non-treated SHR and Wistar rats. *p < 0.05 vs. Wistar group; ^#^p < 0.05 vs. SHR (two-way ANOVA)

As expected, the conscious SHR exhibited high levels of mean arterial pressure (ranging from 162 to 169 mmHg, p < 0.05) compared with the Wistar rats (ranging from 98 to 102 mmHg). Administration of kefir for 7, 15 and 30 days, did not have a significant effect on the high blood pressure, but when it was administered for 60 days a significant decrease in this parameter was observed (145 ± 5 mmHg, p < 0.05). The SHR also exhibited increased resting heart rates (ranging from 376 to 388 bpm, p < 0.05) compared with the Wistar rats (ranging from 335 to 340 bpm). Administration of kefir to SHR for 7, 15 and 30 days did not affect this parameter (Fig. 2b), but after 60 days, it caused a significant decrease in the heart rate of the SHR group (332 ± 15 bpm, p < 0.05).

### Beneficial effects of kefir on the endothelial dysfunction of SHR

**Fig. 3:**
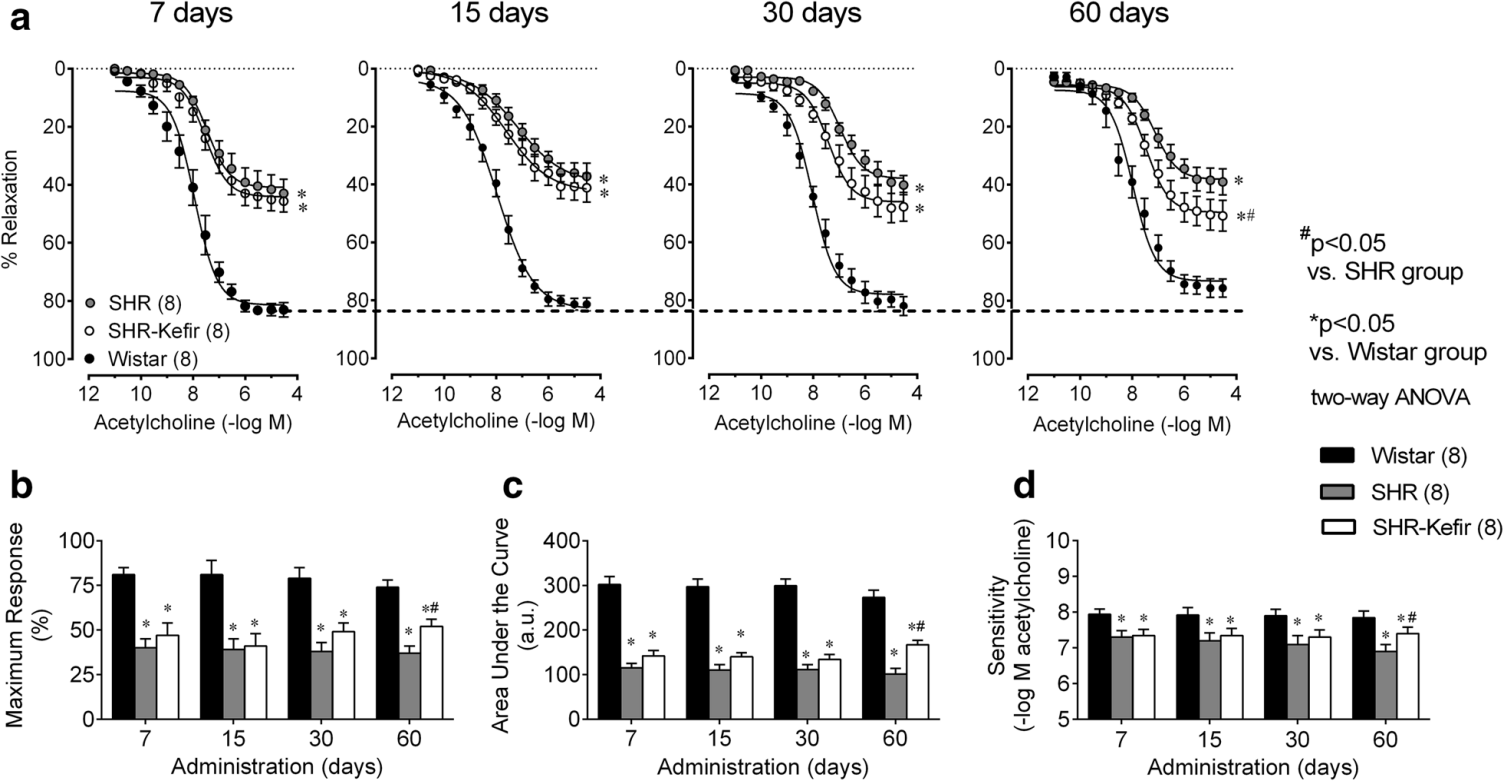
Time-course of the effects of kefir administration on the endothelial dysfunction of SHR. Dose–response curves to acetylcholine-induced relaxations of aortic rings from SHR-kefir compared to the non-treated SHR and to the normotensive Wistar rats (**a**). The* bar graphs* show the maximum relaxation (**b**), the area under the curve (**c**) and the sensitivity (pEC_50_, **d**) to acetylcholine. The values shown are the mean ± SEM. *p < 0.05 vs. Wistar group; ^#^p < 0.05 vs. SHR (two-way ANOVA)

Average values of the vascular endothelium-dependent ACh-induced relaxations of the aortic rings from all groups evaluated are shown as line graphs of Fig. 3. The time-course of the concentration-dependent ACh-evoked relaxation in aortic rings pre-constricted with PE from the normotensive Wistar rats showed a tendency to loss reactivity as indicated by the comparison of the time-points 7 and 60 days (Rmax: 81 ± 4 and 74 ± 4 %, respectively, Fig. 3b). The SHR animals exhibited an impaired relaxation response to ACh (p < 0.01) at all time-points. The three parameters of the dose–response curve to ACh in the untreated SHR showed Rmax (~37 ± 4 %), sensitivity (~6.9 ± 0.2 –log M ACh) and AUC (~101 ± 13 a.u.) significantly impaired when compared with the normotensive Wistar rats (~74 ± 4, ~7.9 ± 0.2 % –log M ACh, and 273 ± 16 a.u, respectively) (Fig. 3a–c). Daily administration of Kefir to the SHR caused a progressive improvement of the vascular responsiveness, reaching significant differences after 60 days of treatment, as noted in the Rmax (52 ± 4 %, p < 0.05), sensitivity (7.5 ± 0.26 –log M ACh, p < 0.05) and AUC (167 ± 10 a.u., p < 0.01) (Fig. 3a–c).

Therefore, the treatment of SHR with kefir for 30 days did not result in significant improvement of the ACh-induced relaxations of aortic rings in all parameters. Interestingly, when the SHR were treated for twice as long, significant effects were observed in all parameters (AUC, Rmax and pEC_50_). Although kefir administration did not restore the values of these three parameters to the same levels observed in normotensive Wistar rats, the beneficial effects on the endothelial dysfunction were highly significant.

### Mechanisms of endothelial dysfunction in the SHR: the role of ROS

**Fig. 4:**
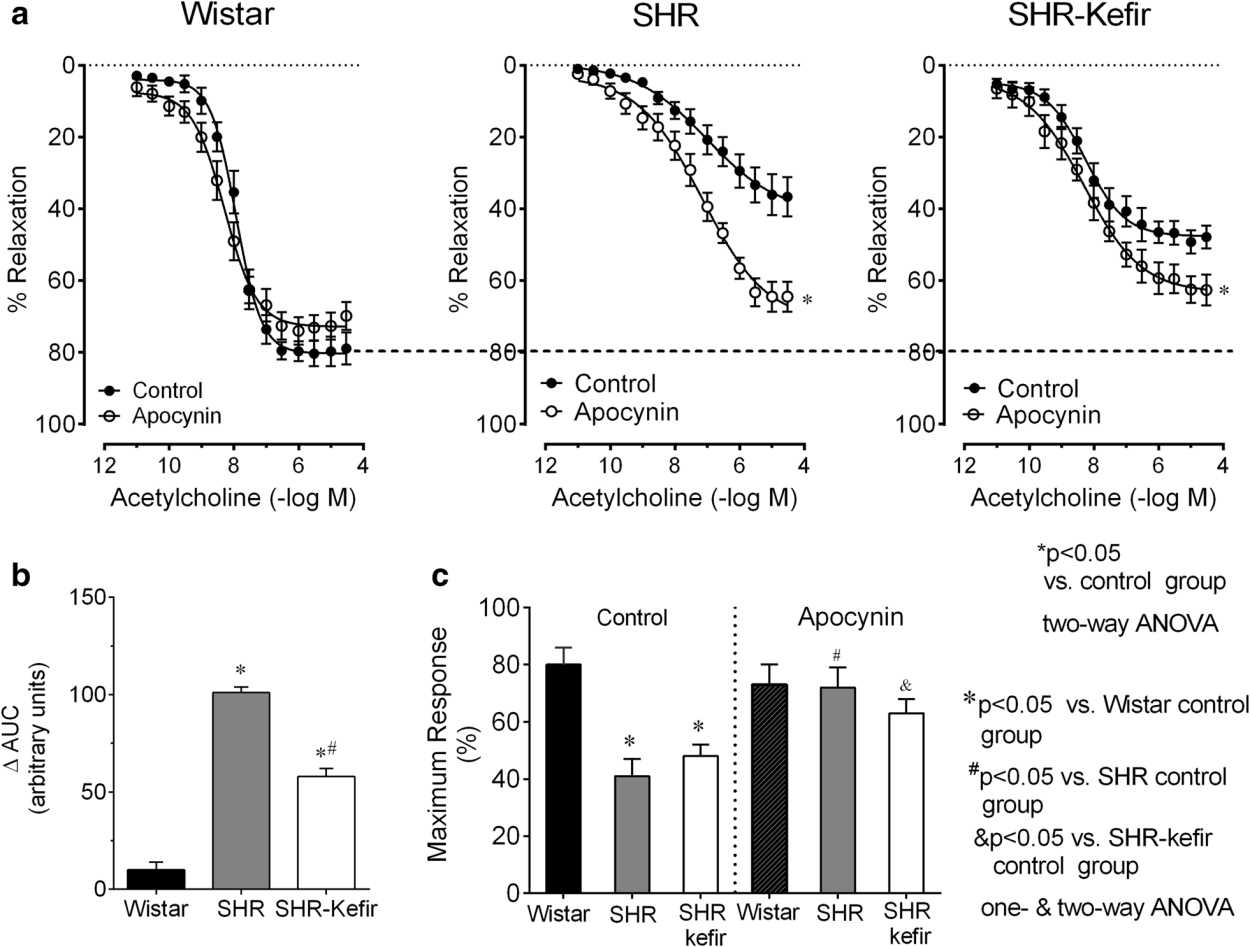
Effects of kefir administration on ROS contribution to the endothelial dysfunction in SHR. Dose–response curves to acetylcholine-induced relaxations of aortic rings from SHR-kefir compared to the non-treated SHR and to the normotensive Wistar rats after the pre-blockade with apocynin (**a**). The* bar graphs* show the difference in the area under the *curve* (**b**) and the maximum relaxation (**c**) in response to acetylcholine. The values are the mean ± SEM (n = 7–8 animals per group)

In a separate set of experiments, we evaluated the contribution of ROS to the impaired endothelium-dependent vasodilator response of the aortic rings to ACh by inhibiting NADPH oxidase with apocynin. Figure 4a, shows the concentration–response curves to ACh with and without inhibition of NADPH oxidase by apocynin in the 3 groups of animals at the time-point of 60 days. The pre-blockade of the aortic rings with apocynin caused a minor effect in the vasodilator response to ACh in the Wistar normotensive rats as shown in the Rmax (−8 %, p > 0.05, Fig. 4c), sensitivity (−9 %, p > 0.05) and ΔAUC (10 a.u., p > 0.05, Fig. 4b). The blockade of the aortic rings with apocynin in the untreated SHR caused a marked exacerbation of the vasodilator response to ACh (Fig. 4a, middle graph); the Rmax changed from 41 ± 6 to 72 ± 7 %, p < 0.05, Fig. 4c), which was very close to Rmax of the Wistar group (81 ± 6 %). Consequently, the difference in the AUC was 101 ± 3 a.u. (Fig. 4b, p < 0.05). On the other hand, in the group of SHR administered with kefir for 60 days, the relaxing response to ACh under the blockade of NADPH oxidase with apocynin was not as exacerbated as it was in the untreated SHR group (Fig. 4a, p < 0.05); the Rmax changed from 48 ± 4 to 63 ± 5 % (Fig. 4c) and the difference in the AUC was 58 ± 4 a.u. (Fig. 4b). The above results indicate that the ROS is the major contributor to the impaired relaxation response to ACh in the SHR and that kefir administration for 60 days partially attenuated this condition because the blockade of NADPH oxidase caused additional improvement in the endothelial function in SHR with Kefir for 60 days.

### Endothelial cell counting and intracytoplasmic ROS production in the aortic arch

**Fig. 5:**
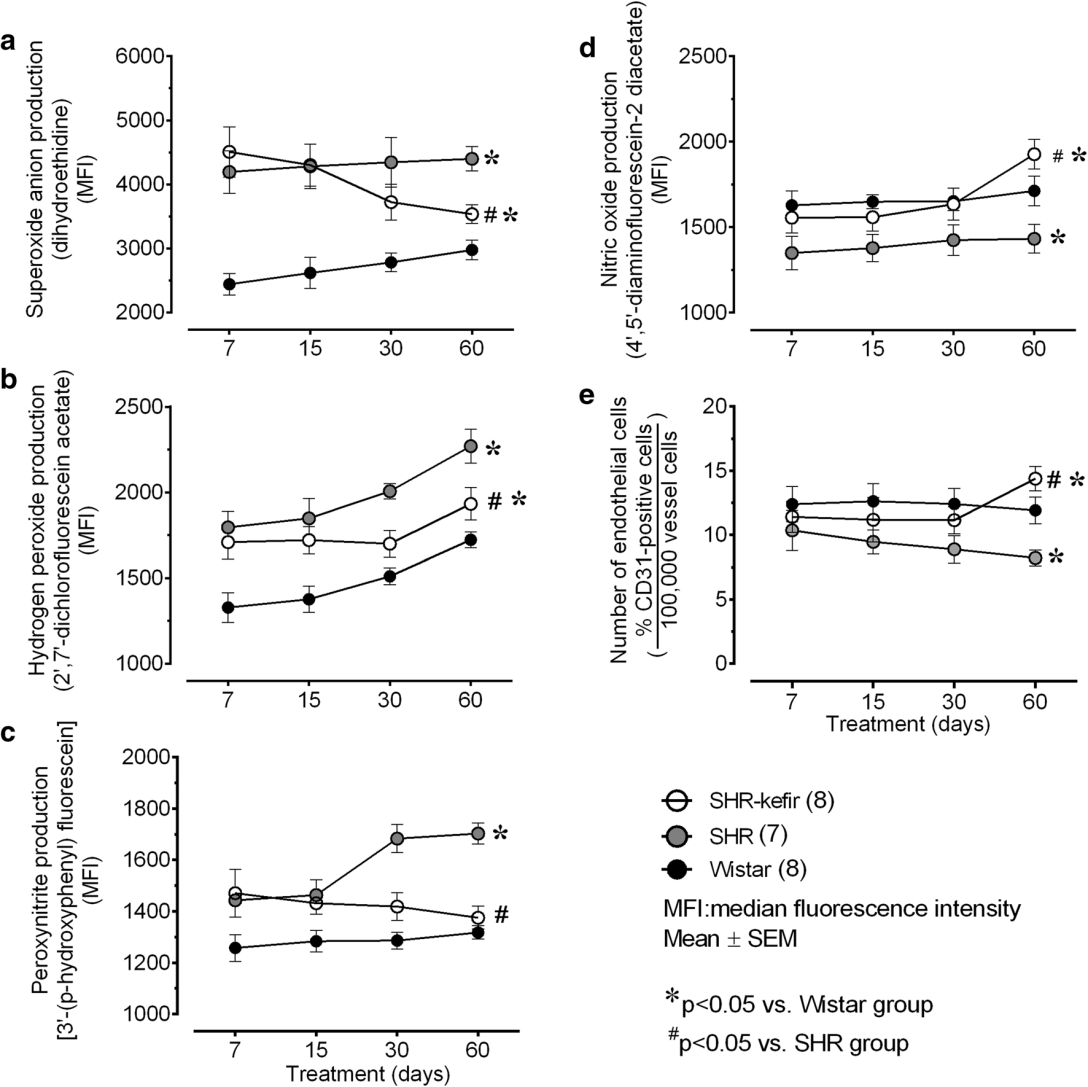
Time-course of changes in the ROS content and the number of endothelial cells in the aorta from the hypertensive rats administered kefir. The* graphs* show production of superoxide anion (**a**), hydrogen peroxide (**b**), peroxynitrite/hydroxyl radical (**c**), nitric oxide (**d**), and the number of endothelial cells (**e**) measured through flow cytometry and comparing SHR-kefir group with non-treated SHR and Wistar rats. The values are the mean ± SEM. *p < 0.05 vs. Wistar group; ^#^p < 0.05 vs. SHR (two-way ANOVA)

To test the anti-oxidative properties of this probiotic, we used flow cytometry to evaluate the production of the three main ROS and to estimate the number of endothelial cells and their NO production in the aortas from the Wistar rats age-matched SHR with or without 7, 15, 30 and 60 days of treatment with kefir. The intracytoplasmic ROS content was determined using the probes DHE, DCF and HPF to quantify the production of •O_2_^−^, H_2_O_2_, and ONOO^−^/•OH^−^, respectively (Fig. 5a–c). A tendency for an increase in the production of •O_2_^−^, H_2_O_2_ and ONOO^−^/•OH^−^ over time was noted, which reached maximum values at 60 days in the Wistar group (2974 ± 151, 1762 ± 22 and 1317 ± 54 MFI, respectively) with significantly higher values being observed in the SHR group (4399 ± 193, 2271 ± 100 and 1702 ± 40, respectively). The time-course effects of the kefir showed a beneficial effect of this probiotic on the production of •O_2_^−^, H_2_O_2_, and ONOO^−^/•OH^−^ in the aortas of SHR when administered for 60 days. As shown in Fig. 5a–c, kefir caused a reduction in the production of •O_2_^−^ (3533 ± 147 MFI, −20 %, p < 0.01), H_2_O_2_ (1933 ± 95 MFI, −15 %, p < 0.05) and ONOO^−^/•OH^−^ (1374 ± 44 MFI, −20 %, p < 0.01) compared with the non-treated SHR.

The flow cytometry approach was also used to evaluate the number of endothelial cells (through CD31-APC) and the production of NO (through DAF) in the aortic arch from the three groups of animals (Fig. 5). The number of aortic endothelial cells was similar in the SHR and Wistar rats at the time-point of 7 days, but the values declined in the SHR and at the time-point of 60 days the number of cells was significantly diminished in this group (33 %, p < 0.05) compared with the Wistar rats (12.9 ± 1.3 CD31-positive cell count). The chronic administration of kefir for 60 days to SHR group abolished this difference in the number of CD31-positive cells in the aorta (Fig. 5e).

As expected, similar results were observed in the analysis of NO production in the aortas of the three groups of animals. The NO production by the endothelial cells isolated from the aortic arch of the SHR was significantly diminished (16 %) compared with the Wistar rats (1712 ± 86 MFI). Kefir administration for 60 days to SHR abolished the difference in the NO production in the aortas from these animals (Fig. 5d).

### Mechanisms of endothelial dysfunction in the SHR: the role of NOS

**Fig. 6:**
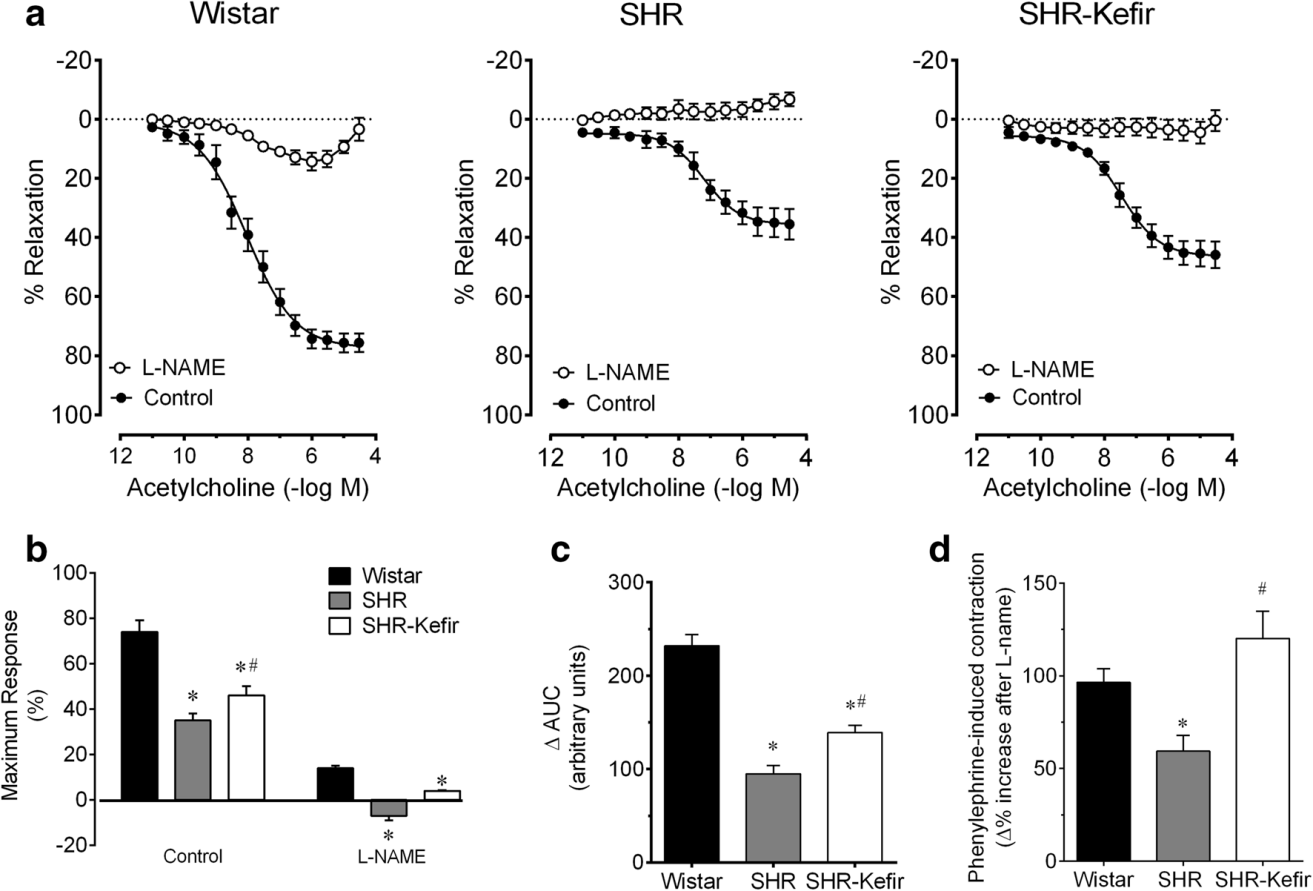
Contribution of the nitric oxide bioavailability to the endothelial dysfunction in SHR administered kefir for 60 days. The* line graph* (**a**) shows the changes in the dose–response to acetylcholine following the endothelial NO synthase blockade with N(G)-nitro-l-arginine methyl ester (L-NAME). The* bar graphs* show the average values of the maximum response (**b**) and the difference in the area under the curve (ΔAUC, **c**) calculated from the dose–response curve obtained during the blockade of the basal NO/cGMP molecular pathway with L-NAME. The* bar graph* **d** shows the basal NO/cGMP molecular pathway activation, indicated by the phenylephrine-induced contraction of the aortic rings during the setting of endothelial NO synthase blockade via L-NAME, comparing the 3 groups of animals. The values are the mean ± SEM (n = 7–8 per group). *p < 0.05 vs. Wistar group; ^#^p < 0.05 vs. SHR [two-way (**a**) and one-way (**b**–**d**) ANOVA]

To address the mechanisms by which kefir produces the beneficial effects on endothelial function, first we investigated the participation of the molecular NO/cGMP pathway in this process by examining the endothelium-dependent vasodilator response of aortic rings to ACh when NOS was inhibited with L-NAME. Figure 6a shows the concentration–response curves to ACh in the presence and absence of inhibition of the NOS pathway by L-NAME in the three groups of animals at the 60-day time-point. Pre-blockade of the aortic rings with L-NAME caused a marked reduction in the vasodilator response to ACh in the Wistar group, reaching an Rmax of 11 ± 1 % (Fig. 6b). The remaining relaxation under the blockade of NOS with L-NAME is attributed to the relaxing factors of the prostanoids pathway, such as the prostacyclin PGI_2_. As expected, the untreated SHR exhibited a contractile effect in response to ACh (Rmax of approximately 7 ± 4 %). The administration of kefir to SHR for 60 days reversed the contraction to a relaxation response (Rmax 3 ± 0.1 %) (Fig. 6b). Consequently, the ΔAUC (difference between before and after L-NAME blockade) was greater in the Wistar rats (257 ± 12 a.u.) than in the SHR (132 ± 9 a.u., p < 0.05) and kefir administration for 60 days caused a significant improvement in the ΔAUC (174 ± 8 a.u, p < 0.05) (Fig. 6c).

The above results indicate that the NO/cGMP pathway is the major contributor to the ACh-induced relaxation response in the normotensive Wistar rat. Our data shows that in the SHR, this pathway seems to be the unique pathway for relaxation and that Kefir administration for 60 days was able to partially repair this abnormal condition. Figure 6d depicts the quantification of the basal NO/cGMP activation estimated through the PE-induced contraction of the aortic rings under the conditions of pre-blockade with L-NAME. Under these conditions, the contraction Rmax was significantly smaller in the non-treated SHR than in the normotensive Wistar rats (59 ± 9 vs. 97 ± 7 %, p < 0.05). Kefir administration for 60 days to SHR caused a marked beneficial effect on this variable (120 ± 14 %, p < 0.05).

### Scanning electron microscopy analysis of vascular endothelial layer architecture

Considering the magnitude of the functional damage observed through the test of the vascular endothelium-dependent relaxation to ACh in the SHR, we used some animals of each group (n = 4 per group) to evaluate the damage to the architecture of the endothelial surface.

**Fig. 7:**
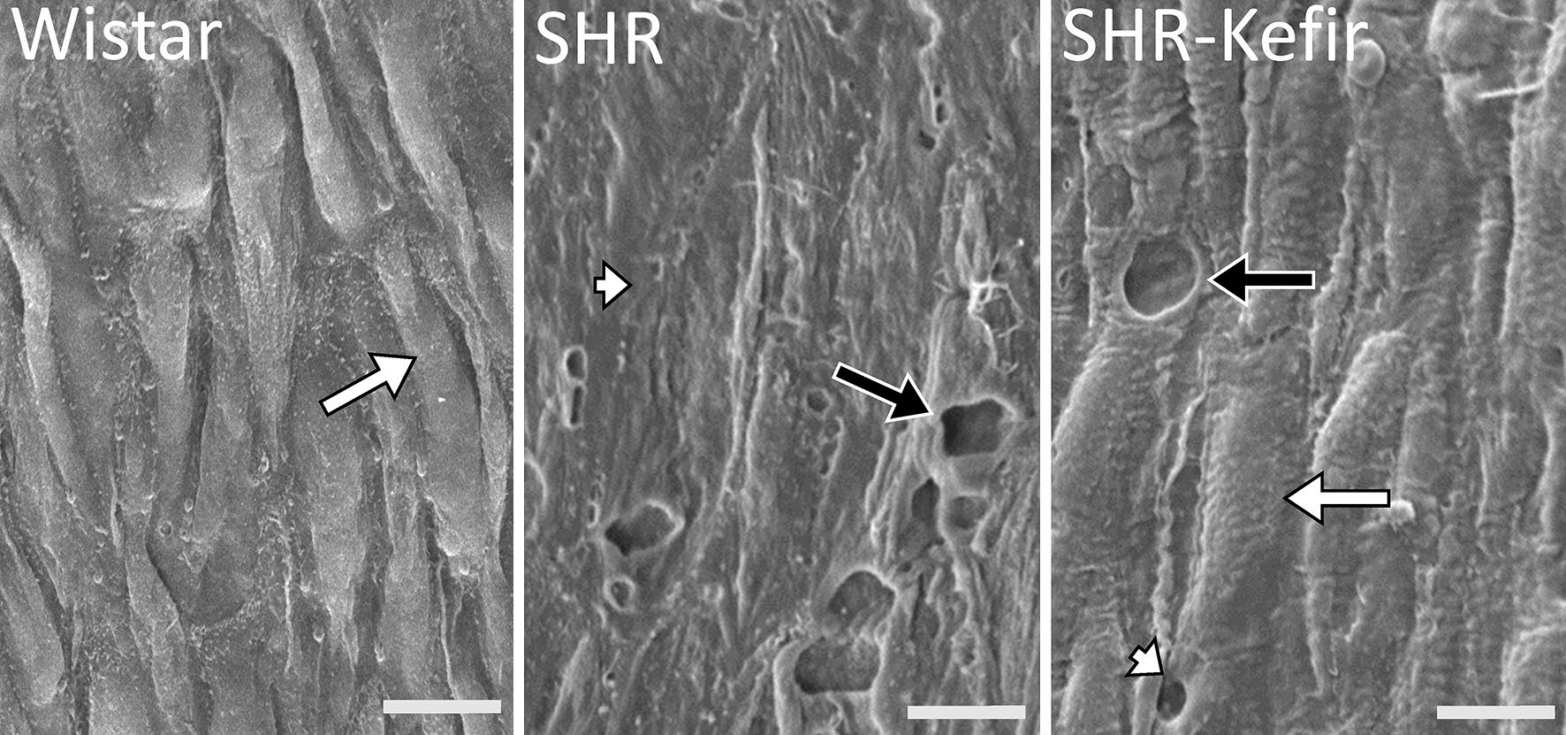
Recovery of vascular endothelial surface architecture in SHR administered with kefir. Scanning electron microscopy showing representative endothelial structure of aortas from a normotensive Wistar rat, a non-treated SHR and a SHR treated with kefir for 60 days. *Scale bar* 10 µm. *White arrow* endothelial cell; *white arrow head* endothelial surface denudation; *black arrow* gaps

En face scanning electron micrographs (Fig. 7) of the thoracic aortas from the normotensive Wistar animals showed a confluent endothelial cell layer. In contrast, those of the SHR exhibit a clear endothelial damage of the luminal surface layer exposing the internal elastic membrane, in which many gaps were observed. Another important finding was that the SHR animals treated with kefir for 60 days exhibited an endothelial surface containing regenerated cells, which partly covered the injured intima area; however, some gaps between the cells still were observed.

### Recruitment of circulating endothelial progenitor cells

Flow cytometric analysis of the EPC was performed using the peripheral blood of Wistar, SHR and SHR-kefir rats. The time-point of 60 days was selected because of the endothelial recovery observed by scanning electron microscopy images. The analyses of the data revealed that the fraction of CD117+/CD31+ double-positive cells in the peripheral blood was significantly reduced in the SHR (86 ± 11, cell count), compared with Wistar group (207 ± 23 cell count, p < 0.01), and kefir administration for 60 days was able to increase the circulating EPC levels (134 ± 7 cell count, p < 0.05).

### Effects of kefir on the endothelium-independent relaxations

The findings of endothelial dysfunction and damage to the endothelial surface in the SHR, led us to evaluate the time-course of responsiveness of the smooth muscle cells to the NO donor SNP in a separate set of experiments, to verify the functional integrity of the smooth muscle cells of the aorta and the effects of kefir administration on these cells.

**Fig. 8:**
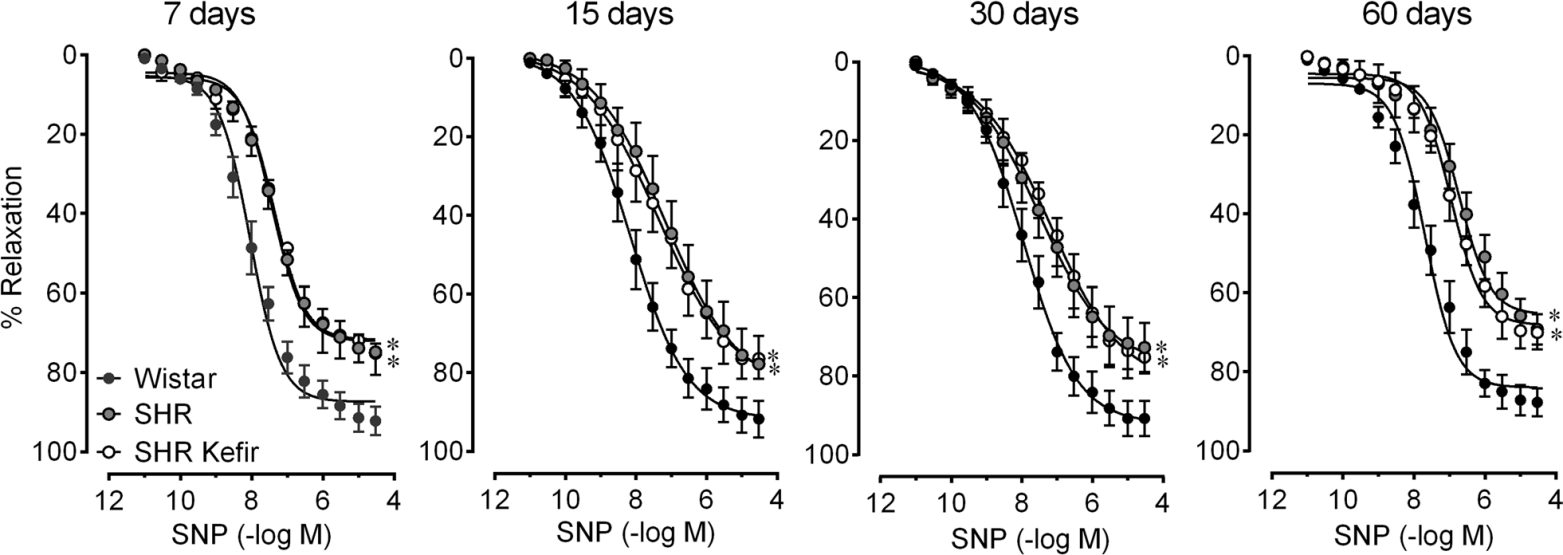
Time-course effect of the kefir administration on the endothelium-independent relation in SHR. The dose–response curves to the NO donor, sodium nitroprusside, of aortic rings from SHR administered kefir compared to the non-treated SHR and to the normotensive Wistar rats (**a**). The values are the mean ± SEM. *p < 0.05 vs. Wistar group (two-way ANOVA)

The dose–response curves for the aortic ring relaxation in response to SNP were impaired in the SHR group compared to those of the Wistar rats. For instance, at the 60-day time-point, the SHR group exhibited a smaller Rmax and sensitivity (72 ± 3 % and 7.4 ± 0.05 –log M) than the Wistar rats (87 ± 5 and 8.0 ± 0.07 % –log M, p < 0.05) (Fig. 8). Our data also showed overlapping dose–response curves from the non-treated SHR and the SHR treated with the probiotic kefir for 7–60 days, i.e., kefir did not cause any effect on the Rmax and or the sensitivity of the aortic rings to SNP at any of the time-points examined.

## Discussion

This study was designed to investigate the effects of kefir on the endothelial dysfunction in the SHR. Using the gold-standard approach to evaluate the vascular reactivity of aortic rings, we observed an impaired relaxation response to ACh in the SHR group, which was mainly due to an imbalance in the NOS/ROS pathways and was accompanied by damage to the endothelial surface layer. The novelty of this study was the demonstration that daily administration of the probiotic kefir for 60 days, caused a significant improvement of the vascular endothelial function. Our data also provide a clear demonstration that the main mechanism for this beneficial effect of kefir involved repair in the vascular endothelial architecture and a reduction of the oxidative stress coupled with an augmentation of the NO bioavailability as well as a simultaneous contribution of EPC recruitment.

Interestingly, our scanning electron microscopy analysis of kefir grains revealed that the majority of the biofilm composed of short and long curved bacilli or ovoid-shaped yeast, consistent with the previous observation of Hamet et al. [19]. Isolates from various traditional Brazilian kefir grains have been identified and characterized as consisting of a matrix made up of polysaccharides/proteins and containing acid bacteria belonging to the genuses *Leuconostoc* spp., *Lactococcus* spp. and *Lactobacillus* spp. and yeast [20]. Accordingly, our microbiological analysis of kefir samples showed the presence of various beneficial bacteria and yeasts, including the specific *Lactobacillus kefiranofaciens* species, which produces kefiran, the main functional component of the beverage [19]. Importantly, in the present study, kefir was produced with kefir grains, which is more desirable than kefir produced with starter cultures [10].

Several studies have suggested that chronic consumption of bioactive fermented milk products may have a protective effect against the development of cardiovascular diseases, including an inversely relationship to the risk of hypertension [9] and hyperglycemia [21]. Kefir has been shown to decrease blood pressure [16] and ischemia–reperfusion injury [11], suggesting that this fermented milk beverage can be useful in the medical management of various cardiovascular conditions. These beneficial effects have been explained by the presence of bacteria and yeast that share a close symbiotic relationship in the kefir grains [13] and result in the production of biogenic elements [19].

Although several studies have demonstrated chronic treatment with bioactive fermented milk products attenuates high blood pressure [8, 9], some have specifically focused on the effects of the complex kefiran in hypertensive subjects [14] and on experimental models of hypertension [15]. Our present data reveal a new important feature of this probiotic in the SHR, the most commonly used model, which may allow that generalization of the results. For instance, we observed that kefir administration lowered the high blood pressure and tachycardia of SHR in a time-dependent manner, reaching the optimal beneficial effects at the time-point of 60 days of treatment. Although arterial hypertension is a multifactorial disease, endothelial dysfunction has been considered to be an important contributor to and thus a valuable biomarker for hypertension and other cardiovascular diseases, highlighting the importance of our analysis of the effects and mechanisms of kefir on the aortic rings from SHR.

Very few studies have been designed to evaluate the beneficial effects of the consumption of fermented milk with bioactive components on endothelial dysfunction in SHR, and their results indicated opposite effects [9]. Our data demonstrated that the endothelium-dependent relaxation of aortic rings in response to ACh was significantly smaller in the SHR compared with normotensive Wistar rats, corroborating some previous studies [22, 23] but in disagreement with others [24]. Considering that our focus was to study the vasodilation process and based on the observation by others [23] that very high doses of ACh in aortic rings from the SHR induce endothelium-dependent contractions, the maximal dose of ACh tested in the present experiment was less than 10^−4^ mol/L. In addition, our first novel finding consisted of the demonstration that kefir administration caused a time-dependent improvement in the endothelial function in SHR, very similar the observed improvements in blood pressure and heart rate. Considering the similar time-course of the beneficial effects on hypertension and endothelial dysfunction, the idea that the improvement of endothelial function may have contributed to the attenuation of arterial hypertension in the SHR to which kefir was administered seems reasonable.

Experimental genetic models of arterial hypertension are characterized by endothelial dysfunction, which is a primary contributor to the increased generation of ROS, leading to NO breakdown [1, 22, 25]. Therefore, we first focused on the contribution of ROS to the endothelial dysfunction observed in the SHR model and on the potential of chronic administration of kefir to ameliorate this dysfunction. The endothelial dysfunction occurs as consequence of an exaggerated production of •O_2_^−^, which is mainly derived from NADPH oxidase and which scavenges NO to form peroxynitrite and consequently decreases the NO bioavailability in the vascular endothelium [4, 26]. This explains the enhanced generation of NADPH-mediated ROS in the aorta of SHR [27].

Our data showed that the blockade of the main source of ROS with apocynin was ineffective in rings from the normotensive Wistar rats but significantly improved the blunted endothelium-dependent relaxation ACh in aortic rings from the SHR. Our results are in agreement with others in showing a relationship between hypertension and oxidative stress and between increased ROS production and impaired vascular reactivity [28, 29]. Moreover, we had previously demonstrated that therapies that decrease ROS production in endothelial cells from hypertensive and atherosclerotic mice result in the reestablishment of the vascular function [3, 18, 30]. Importantly, in this experiment, the SHR animals that were chronically treated with kefir exhibited normalized relaxation response to ACh, and also a very small change in the dose–response curve to ACh under the apocynin blockade, which was comparable to that observed in the normotensive Wistar rats. The present data support other studies that had demonstrated that kefir treatment of rats and humans reduced the risk of developing hypertension-associated complications [31]. Recently, others have also observed an improved vascular reactivity associated with the reduction of oxidative stress in human beings and animals with metabolic disturbances when treated with probiotic *Lactobacillus* [32].

The cumulative data, including those from the present study and from other models of experimental hypertension, are consistent with the concept that oxidative stress contributes to cardiovascular complications of hypertension and endothelial dysfunction and vice versa [3, 18, 33]. In this context, our hypothesis was that the probiotic kefir could reduce oxidative stress and consequently increase NO availability in the aortic endothelial cells as an additional mechanism, contributing to beneficial cardiovascular effects of nutritional therapy. In agreement with the vascular reactivity, the aortic endothelial cells from SHR exhibited an excessive production of ROS (mainly ONOO^−^/•OH^−^) compared with the cells from the Wistar rats, which was attenuated by kefir treatment for 30 and strongly decreased by treatment for 60 days. Importantly, kefir treatment increased NO availability and the number of EPC counted by flow cytometry in the aortic tissue of SHR, confirming the concept that endothelial function is restored by this probiotic. Moreover, we also used pharmacological approaches to evaluate whether kefir could improve the NO/cGMP signaling.

The blockade of NOS with L-NAME markedly reduced the relaxation response to ACh of aortic rings from Wistar rats (~70 %), indicating that most of the relaxation is dependent on this pathway. In contrast, L-NAME abolished the ACh-induce relaxations and evoked concentration-dependent contractions in the non-treated SHR, indicating that the reduced (~35 %) relaxation response to ACh might be attributed to the NOS pathway. In the SHR treated for 60 days with kefir, our data also showed that L-NAME completely blocked the improved vasodilation responsiveness, which indicates that the improvement of endothelial function in SHR involved (at least in part) the restoration of NO bioavailability. Interestingly, our data corroborates recent findings which demonstrate that the anti-oxidative properties of kefir involve the activation of scavenging enzymes and/or decrease expression/action of other pro-oxidant sources enzymes [34].

It is noteworthy that under the blockade of the NOS pathway, ACh still caused a residual vasodilation (~10 %) in the Wistar rats and a contraction (~10 %) in the SHR, whereas neither significant contraction nor vasodilation was observed in the aortic rings from the SHR treated with kefir. Thus, the remaining relaxation induced by ACh after incubation with L-NAME in Wistar rats could be attributed to a vasodilator prostanoid pathway (e.g., PGI_2_). On the other hand, the ACh-induced contractions of aortic rings under the L-NAME blockade in SHR could be the result of an ACh-induced increase in the levels of vasoconstrictor prostanoids (e.g., endoperoxides, thromboxane A_2_ and prostaglandin E_2_), which might explain that response [25]. This is also supported by the finding that incubation with L-NNA and indomethacin together completely prevented the ACh-induced contraction in the SHR model [23].

Based on the experimental evidence, it is possible that the augmented ROS production (as observed in the SHR) could inactivate NO in the endothelial cells, causing both endothelial dysfunction and diminishing EPC mobilization and function [35]. Thus, an attempt was also made to gain insights into the structural changes in the endothelial surface of the aorta. This possibility led us to examine the endothelial surface layer using scanning electronic microscopy. As expected, in conjunction with our functional data, the substantial re-endothelialization we observed in SHR rats treated with kefir for 60 days could explain the partial recovery of the architecture surface, with a simultaneous increase in endothelial cell counts in this group. These observations are consistent with the results obtained in the present study with respect to the vascular reactivity and in ROS production, suggesting that kefir presented a prompt and notable re-endothelialization action. Taken together, so far our results allow us to conclude that, chronic treatment of SHR with kefir, in addition to the reduction of ROS generation and, subsequently, restoration of the NO availability in the aorta also partially repairs the damage to the endothelial surface layer in that group. The increases in the endothelial cell count and re-endothelialization in the hypertensive rats chronically treated with kefir led us to design a new protocol focused on the EPC. On the basis that NO plays an important role in the EPC mobilization [36, 37], re-endothelialization in the SHR chronically treated with kefir could be attributed to the increased production of NO by the endothelial cells, which could attract EPC to the injured artery layer. In addition, it has been previously demonstrated that hypertensive subjects exhibit diminished number of functional circulating EPC [25], consistent with our findings in the SHR group. However, we demonstrated that SHR rats treated with kefir for 60 days exhibit a marked increase in the circulating EPC numbers.

The interplay between oxidative stress and inflammation directly influence EPC mobilization [35] and indirectly lead to endothelial surface denudation [38], contributing to the development of hypertension [39], consistent with our data in the SHR animals. There is evidence that EPC may act on the oxidative stress by diminishing deleterious intracytoplasmic ROS and simultaneously augmenting NO [36, 37]. In addition, others have shown that the increased NO release is due to enhanced activity of NO synthase [40] and these effects could in turn induce EPC mobilization from the bone marrow to the peripheral blood [41]. Once mobilized into the peripheral circulation, EPC locate to the injured parts of the artery and are involved in the re-endothelialization [42–44]. Therefore, the novelty of the present study is that kefir chronically administered to SHR resulted in a significant increase in the EPC recruitment, which could explain, at least in part, the notable reparative effect of this beverage on the architecture of the endothelial surface and in the improvement of endothelial function in SHR treated with kefir for 60 days. Accordingly, our data provide the first evidence that the beneficial effects of kefir on the endothelial dysfunction in hypertensive animals may be linked to the EPC recruitment.

A controversial issue in endothelial dysfunction in the SHR model is the functional responsiveness of the vascular smooth muscle cells. Accordingly, impairment of the relaxation caused by the direct smooth muscle vasodilator SNP in SHR has been reported by some investigators [26, 45] but not by others [46]. In the present study, the time-course evaluation of aortic rings of the SHRs showed significantly smaller relaxation in the SNP than those of age-matched Wistar rats, at all time-points. These results suggest that arterial hypertension in this model is also associated with a defect in smooth muscle relaxation. It is noteworthy that kefir treatment even for 60 days did not significantly affect the endothelium-independent relaxation in SHR. This result indicates that the beneficial effects we observed of kefir on the endothelial dysfunction might be limited to the endothelial surface cells.

## Conclusions

**Fig. 9:**
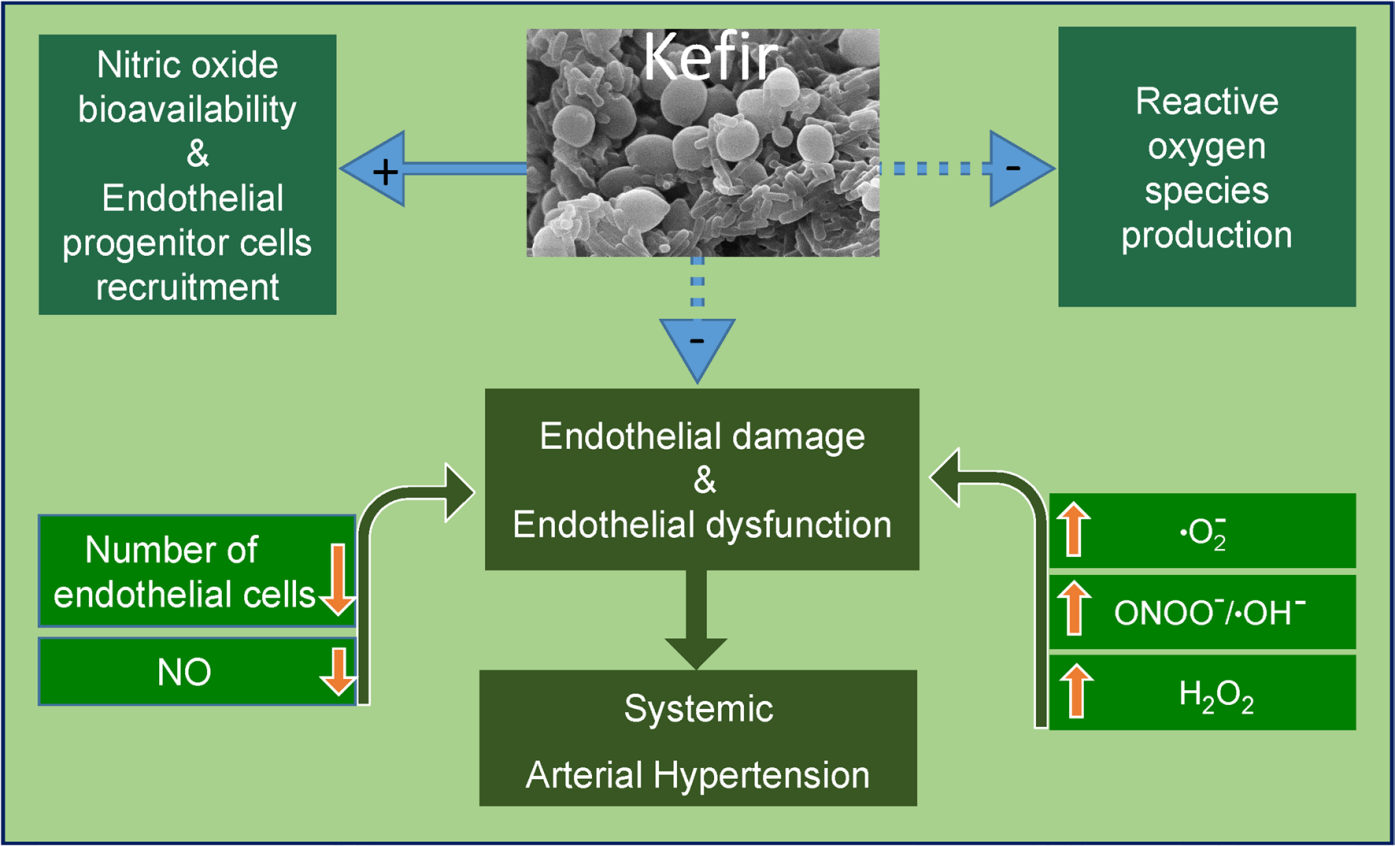
Beneficial effects of kefir in arterial hypertension. Simplified scheme of main effects of chronic administration of kefir on the endothelial dysfunction in SHR

Taken together, our data provide the first evidence that kefir (even at a low dose) was able to attenuate endothelial dysfunction in the large vessels in SHR by decreasing intravascular ROS production and consequently restoring intravascular NO availability. Additionally, kefir also seems to play a relevant role in the re-endothelialization of the vasculature, probably by EPC recruitment and this may contribute for the (partial) repair of the vascular endothelial architecture in the SHR. Figure 9 summarizes the beneficial effects of kefir on the endothelial dysfunction based on our data in the present study with SHR.

## Perspectives

The present data corroborates the idea that kefir could be used to design translational plans for treatment and/or prevention of cardiovascular diseases in which beneficial microorganisms delay the progression of disease. It is possible that the beneficial effects of kefir on the oxidative stress of hypertensive animals may be associated with some bacteria strains that diminished the levels of circulating pro-inflammatory cytokines, suggesting that some specific strain of kefir has antioxidant and anti-inflammatory properties. Studies performed in animals fed with isolated bacteria from kefir grains, such as *Lactobacillus kefiranofaciens*, which was an important component of kefir grains in our study, presented beneficial cardiovascular effects in SHR [16, 47]. Therefore, the future challenge is to identify components of kefir grains to improve endothelial function in cardiovascular diseases, favoring the growing field of development of functional foods.

## Abbreviations

ACh: Acetylcholine
ΔAUC: Difference in the area under the curve
DAF: Diaminofluorescein
DCF: Dichlorofluorescein
DHE: Dihydroethidine
EPC: Endothelial progenitor cell
H_2_O_2_: Hydrogen peroxide
HPF: Hydroxyphenyl fluorescein
−log EC_50_: Sensitivity
L-NAME: *N*(G)-nitro-l-arginine methyl ester
MFI: Median fluorescence intensity
NO: Nitric oxide
•O_2_^−^: Superoxide anion
ONOO^−^/•OH^−^: Peroxynitrite/hydroxyl radical
PE: Phenylephrine
Rmax: Maximum response
ROS: Reactive oxygen species
SHR: Spontaneously hypertensive rats
SNP: Sodium nitroprusside
